# Acoustically driven electromagnetic radiating elements

**DOI:** 10.1038/s41598-020-73973-6

**Published:** 2020-10-12

**Authors:** Ahmed E. Hassanien, Michael Breen, Ming-Huang Li, Songbin Gong

**Affiliations:** 1 Micro and Nanotechnology Laboratory, Department of Electrical and Computer Engineering, University of Illinois at Urbana-Champaign, Urbana, IL 61801 USA; 2 Department of Power Mechanical Engineering, National Tsing Hua University, Hsinchu, 30013 Taiwan

**Keywords:** Electrical and electronic engineering, Mechanical engineering, Applied physics

## Abstract

The low propagation loss of electromagnetic radiation below 1 MHz offers significant opportunities for low power, long range communication systems to meet growing demand for Internet of Things applications. However, the fundamental reduction in efficiency as antenna size decreases below a wavelength (30 m at 1 MHz) has made portable communication systems in the very low frequency (VLF: 3–30 kHz) and low frequency (30–300 kHz) ranges impractical for decades. A paradigm shift to piezoelectric antennas utilizing strain-driven currents at resonant wavelengths up to five orders of magnitude smaller than electrical antennas offers the promise for orders of magnitude efficiency improvement over the electrical state-of-the-art. This work demonstrates a lead zirconate titanate transmitter > 6000 times more efficient than a comparably sized electrical antenna and capable of bit rates up to 60 bit/s. Detailed analysis of design parameters offers a roadmap for significant future improvement in both radiation efficiency and data rate.

## Introduction

Portable wireless devices have become ubiquitous over the last decade, and with the growth of the internet of things (IoT), demand for small, efficient wireless communication systems continues to accelerate. While the development of wireless systems has kept pace with demand at higher frequencies, implementation of portable low-frequency systems has remained impractical for nearly a century. Extreme inefficiency as electrical antennas are scaled to compact sizes and limited data rates due to limited antenna bandwidths have resulted in mobile wireless communication to be developed almost exclusively at much higher frequencies. Compact antennas at the very high frequency (VHF 30–300 MHz) and ultra-high frequency (UHF 300–3000 MHz) are well developed and suited for transmitting data at high bit rates. However, increased spectral crowding and relatively large propagation loss in those bands make them unsuitable for widespread arrays of remote, low power sensors in rural areas or long-range communication elements over rugged terrain desirable for IoT or defense applications^1^. Compared to VHF and UHF signals, radiation at the ultra-low (ULF 0.3–3 kHz) and very low frequency (VLF 3–30 kHz) ranges exhibits relatively low propagation loss, enabling communication underwater up to 20 m^2^ and through hundreds of feet of earth^3^. Additionally, VLF radiation can propagate as ground waves that reflect back and forth between the Earth's surface and ionosphere with very low atmospheric attenuation of ~ 2–3 dB/100 km^4^. Such propagation properties make VLF communication well suited for applications where distance or line-of-sight limitations make higher frequency portable systems unviable and high data rates are less valuable than reliable, long-distance communication. Development of sensor arrays such as remote moisture detectors spread over large forested areas to detect wildfires or emergency text or voice communication in inaccessible terrain benefit greatly from operating at VLF frequencies without being capped by the limited bandwidth. However, while the desirable propagation properties ensure continued demand for portable, long-range VLF transmitters, use of VLF antennas has been largely restricted to submarines^5,6^ and large base installations^7,8^ such as the VLF transmitter in Cutler, Maine.

Despite decades of investigation, compact antennas in the VLF and low frequency (LF 30–300 kHz) bands have remained an unattainable holy-grail considered impractical due to the fundamental tradeoff between antenna efficiency and electrical size. Efficient electrical antennas require operation near electromagnetic resonance, typically restricting the physical size to be larger than one-tenth of a wavelength λ/10. Fundamental analysis on the tradeoff between antenna size and efficiency was first conducted by Wheeler^9^ and Chu^10^ in the 1940s and extensive subsequent work^11–13^ defined the size limit for an efficient electrically small antenna (ESA). Decreasing the size of an ESA below that limit results in a diminished radiation resistance, which leads to a low radiation efficiency as resistive losses begin to dominate^14^. Furthermore, as the size of electrical antennas becomes much smaller than λ, the reactive component of the antenna impedance becomes increasingly large. The small radiation resistance in conjunction with the much larger antenna reactance results in a large impedance mismatch with the driving electronics. Tuning out the large reactance to improve the matching efficiency requires an impedance matching circuit, but for frequencies below 1 MHz, the large size and lossy nature of the matching circuit have made ESAs impractical to implement.

Recently, piezoelectric resonant acoustic antennas have been considered to surpass the inefficiency of ultra-sub wavelength ESAs required for portable VLF communication. First proposed by Mindlin^15^, piezoelectric transmitters couple mechanical vibration into electrical radiation. Acoustic waves propagate at velocities 10^5^ times lower than electromagnetic waves, enabling resonant operation for mechanical antennas at frequencies five orders of magnitude lower than similar sized electrical counterparts. Resonant impedances of acoustically driven antennas can be easily matched to driving electronics, removing the need for bulky, inefficient matching circuits. More recently, additional studies on the radiation properties of piezoelectric antennas^16,17^ and early prototypes at both UHF^18,19^ and VLF^20^ have been demonstrated to show promise as compact antennas with efficiency advantages over ESA.

In this paper, we demonstrate an acoustically driven and modulation inducible radiating element (ADMIRE) using lead zirconate titanate (PZT) as the piezoelectric material which redefines VLF transmitters by exceeding the matched efficiency of ESAs by orders of magnitude and demonstrating novel shaping of near and far-field regions using high-permittivity materials. While the presented matched antenna efficiency is already more than 6000 × that of an equivalently sized ESA, it is still far from the anticipated limits of piezoelectric antennas. A full analysis of the design space for piezoelectric antennas is detailed, paving the way for the subsequent development of compact, high-efficiency piezo-transmitters with the potential for widespread use in low-frequency wireless communication systems.

### Theory

Acceleration of charges, including dipole moment flipping, results in far-field electromagnetic (EM) radiation with field components that are inversely proportional to the distance traveled away from the radiating element^21^. Using this concept, any element that contains a time-varying dipole moment, such as the acoustically excited piezoelectric materials described in this paper, can be considered a radiating element. Piezoelectric materials lack inversion symmetry within their crystalline structure, resulting in a linear coupling between the electrical and the mechanical domain parameters via the reversible piezoelectric effect. In particular, the direct piezoelectric effect is the electrical polarization produced by an applied mechanical stress^22^. For a time-varying stress, radiation is produced with the time-varying electrical polarization.

**Figure 1:**
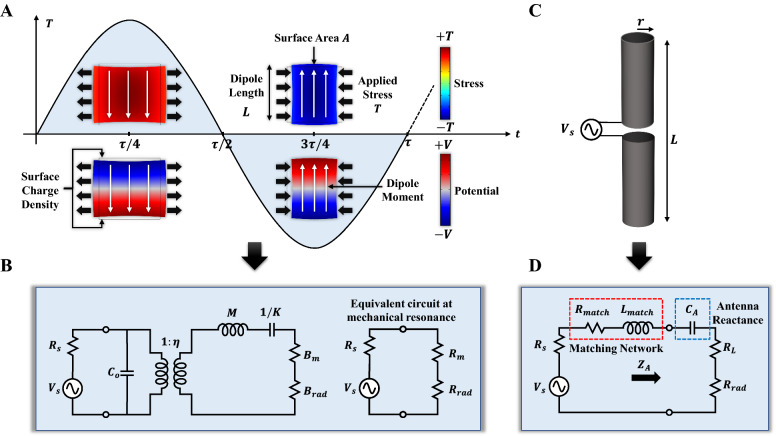
Comparison between acoustically driven and modulation inducible radiating elements (ADMIREs) and electrically small antennas (ESAs). (**A**) ADMIRE-conceptual diagram with a sinusoidal time-varying mechanical stress applied, resulting in time-varying electrical polarization. (**B**) Butterworth Van-Dyke equivalent circuit model of the ADMIRE. (**C**) ESA representation (infinitesimal dipole). (**D**) ESA equivalent circuit model at low frequencies (< 1 MHz) with large antenna reactance dominating the antenna input impedance which requires an impractical matching network.

This concept is further explained in Fig. 1A, where a sinusoidal force, with period $\tau$, is exerted on a piezoelectric material resulting in electric polarization with surface charge density ${\sigma }_{q}$ which can be calculated using the piezoelectric constitutive equations as follows^17^:

1 $${\sigma }_{q}=dT=d{C}^{E}S,$$

2 $$I={\sigma }_{q}A\omega =d{C}^{E}SA\omega ,$$

where $d$ is the piezoelectric strain constant, $T$ is the applied stress, ${C}^{E}$ is the stiffness at constant electric field and $S$ is the resulting strain. Equation (1) assumes an average strain throughout the entire volume of the piezoelectric material for simplicity, which provides the effective modal response in the dynamic excitation case. The effective dipole current is calculated in Eq. (2), where $A$ is the surface area of the accumulated charges and $\omega$ is the angular frequency of the applied stress. The generated magnetic field density in the far-field region due to the dipole current is then formulated as^21^:

3 $$\left|{B}_{far}\right|=\frac{{\sigma }_{q}A}{4\pi {\varepsilon }_{o}}\frac{L{\omega }^{2}}{{c}^{3}R},$$

where $L$ is the dipole moment length, ${\varepsilon }_{o}$ is the permittivity of the free space, and $c$ is the speed of light. The corresponding far-field electric field $\left|{E}_{far}\right|=c\left|{B}_{far}\right|$.

For comparison, both the ADMIRE and a generic electrically small antenna (ESA), are shown in Fig. 1A,C, respectively. Both types of antennas can be modeled in the electrical domain with the equivalent circuit representations shown in Fig. 1B,D, respectively. The ADMIRE is modeled with the Butterworth Van Dyke (BVD) model^23^. The electrical equivalent circuit model for ADMIREs is composed of a capacitor ${C}_{o}$, a transformer with turns ratio $\eta$, an inductor $M$, a capacitor $1/k$, and a resistor ${B}_{m}$ representing the static capacitance between the input terminals, electromechanical transformation ratio, mass, mechanical stiffness, and mechanical damping, respectively. Another resistor ${B}_{rad}$ is added to represent the losses due to EM radiation. This model can be further simplified by eliminating the transformer. In the simplified model, ${R}_{m}$ represents the mechanical losses, ${R}_{rad}$ represents the radiation losses, ${\omega }_{r}$ is the resonance frequency, ${L}_{m}$ is the motional inductance representing the mechanical mass effect, and ${C}_{m}$ is the motional capacitance representing the mechanical stiffness effect. At the mechanical resonance the reactive components cancel out (${{\omega }_{r}L}_{m}=1/{{\omega }_{r}C}_{m}$) and the input impedance of the ADMIRE is ${R}_{m}+ {R}_{rad}\ll 1/{\omega C}_{o}$ where ${R}_{rad}\ll {R}_{m}$. The BVD circuit parameters can be calculated from the following equations^24^:

4 $${R}_{m}=\frac{{\pi }^{2}}{8{\omega }_{r}{C}_{o}{k}_{t}^{2}Q}, {L}_{m}=\frac{{\pi }^{2}}{8{\omega }_{r}^{2}{C}_{o}{k}_{t}^{2}}, {C}_{m}=\frac{8}{{\pi }^{2}}{C}_{o}{k}_{t}^{2},$$

where ${k}_{t}^{2}$ is the electromechanical coupling coefficient and $Q$ is the mechanical quality factor. The BVD parameters can be obtained by fitting the equivalent circuit response to the finite element modeling (FEM) result as shown in Fig. 3E where the measurement, FEM, and parameters required to calculate circuit components are reported. As previously explained, ESAs at low frequencies (< 1 MHz) have a large reactive element that requires impractical matching compared with the ADMIRE which is designed to be impedance matched.

It can be shown that the ADMIRE radiation efficiency, defined as the radiated power divided by the input power, is proportional to the piezoelectric material properties and dimensions. For radiation efficiencies much less than one, the radiation efficiency can be written as^17^:

5 $${\xi }_{ADMIRE}\propto {d}^{2}{C}^{E}VQ{\omega }^{3},$$

where $V=LA$ is the volume of the ADMIRE. The relative radiation efficiency for similarly sized ADMIRE and ESA can be formulated as^17^:

6 $${\xi }_{rel}=\frac{{\xi }_{ADMIRE}}{{\xi }_{ESA}}\propto \frac{{d}^{2}{C}^{E}Q\omega }{{\sigma }_{c}},$$

where ${\sigma }_{c}$ is the electrical conductivity of the ESA metallic material. From Eq. (6), the relative radiation efficiency of the ADMIRE can be increased by selecting a material with larger stiffness, quality factor, and especially the piezoelectric strain constant due to its squared behavior. However, the main advantage of mechanical antennas arises from the mismatch efficiency of ADMIREs compared to ESAs at low frequencies below 1 MHz. The typical efficiency definition is the ratio of radiated power to the input power (${P}_{rad}$/${P}_{in}$). In this paper, we deviated from this definition to account for not only efficient radiation but also maximum radiated power. We define the matched antenna efficiency as the ratio of radiated power to the maximum power available for radiation from the source, which is achieved at conjugate matching for a lossless antenna.While ADMIREs can be designed to have real resonant impedances that achieve high matching efficiency without the need for a matching network at low frequencies, ESAs are well known to exhibit small radiation resistances and large reactive components which result in an enormous mismatch efficiency (very low matched total antenna efficiency). To improve the matched antenna efficiency, ESAs require bulky impedance matching circuits to tune out the reactive component. The relative matched antenna efficiency of the ADMIRE, normalized with respect to an impedance-matched ESA can be expressed as:

7 $${\xi }_{tot}^{rel}=\frac{{\xi }_{tot}^{ADMIRE}}{{\xi }_{tot}^{ESA}}=\frac{{R}_{rad}^{ADMIRE}}{{R}_{rad}^{ESA}}\frac{{\left({R}_{rad}^{ESA}+{R}_{loss}+{R}_{match}+{R}_{s}\right)}^{2}}{{\left({R}_{rad}^{ADMIRE}+{R}_{m}+{R}_{s}\right)}^{2}},$$

where ${R}_{rad}^{ADMIRE}$ and ${R}_{rad}^{ESA}$ are the ADMIRE and the ESA radiation resistances respectively, ${R}_{loss}$ is the ESA conduction/dielectric losses, ${R}_{match}$ is the matching resistance resulting from the finite quality factor of the matching inductor, and $R_{s}$ is the source resistance as shown in Fig. 1. Even with matching networks for the ESAs, typically consisting of low-frequency inductors with quality factors less than a few hundred, the matched impedance seen by the source remains in the kilo-ohms range, resulting in matched antenna efficiencies more than 6400 × greater in favor of ADMIREs over ESAs (more information can be found in the Supplementary document).

In addition to the material properties essential for efficient radiation, the relative permittivity of the piezoelectric material bears crucial consideration for reliable antenna operation. As the bound charge densities on the top and bottom surfaces of the ADMIRE are flipped to induce the dipole current for radiation in Eq. (2), an electric field $E$ is produced. This electric field is inversely proportional to the relative permittivity as shown in Eq. (8):

8 $$E\propto \frac{{\sigma }_{q}}{{\varepsilon }_{r}{\varepsilon }_{o}},$$

where ${\varepsilon }_{r}$ is the relative permittivity of the piezoelectric material. The radiated field strength for an antenna is determined by the maximum achievable current and its distribution. In the case of ADMIREs, the maximum current limit is determined by the charge density that results in electric near-fields just below the breakdown limit of the surrounding environment. The electrical breakdown of the surrounding material puts a maximum limit on the achievable charge density (${\sigma }_{q}<{\varepsilon }_{r}{\varepsilon }_{o}{E}_{Breakdown}$) leading to a maximum limit on the radiated power. The total radiated power in watts can be derived via integrating the Poynting vector over a sphere in the far-field which gives ${P}_{rad}={\left({\sigma }_{q}AL{\omega }^{2}\right)}^{2}/6\pi {\varepsilon }_{o}{c}^{3}$, therefore, a high relative permittivity piezoelectric material is needed to maximize the radiated power without electrically breaking down the surrounding medium. The maximum radiated power that can be achieved will depend on material properties, as well as the antenna dimensions and operating frequency.

**Figure 2:**
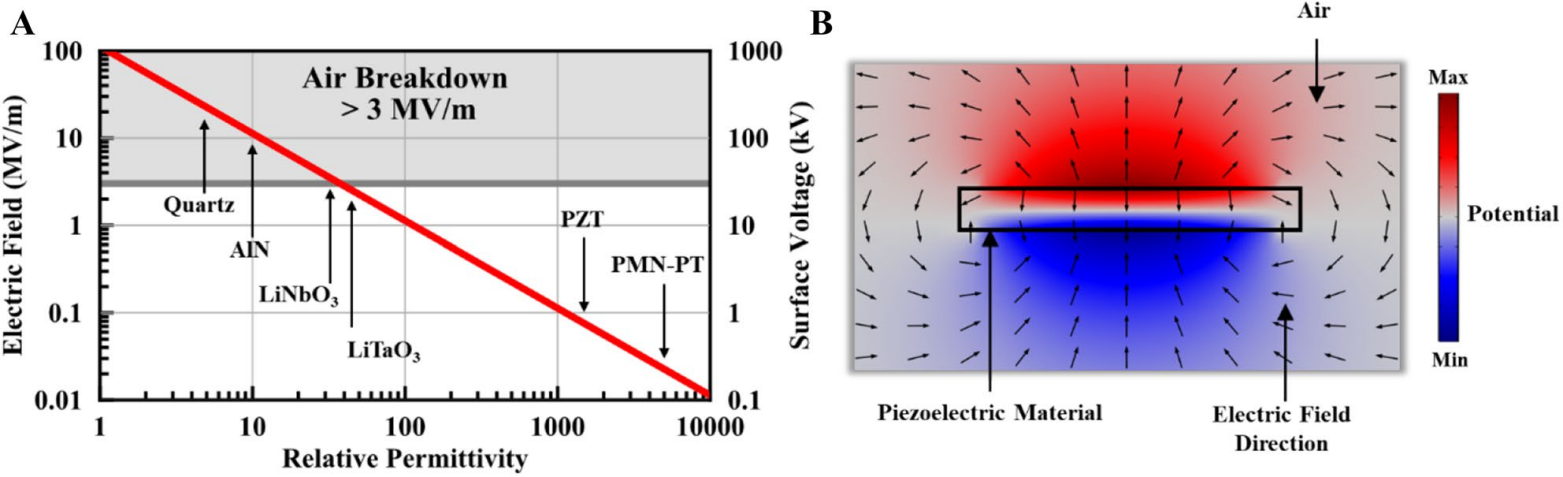
Comparison of electrical polarization response for different piezoelectric materials. All materials are assumed to have a charge density of ± 1 mC/m^2^ on the top and bottom surfaces with a thickness (distance between surfaces) of 1 cm. (**A**) The electric field and the corresponding surface voltage versus the piezoelectric material relative permittivity. The top gray region is the air breakdown region where the electric field exceeds 3 MV/m. (**B**) The voltage distribution due to the electric polarization and the electric field direction represented by the black arrows (simulated with COMSOL Multiphysiscs v4.4).

Figure 2A compares a few commonly used piezoelectric materials with different values of relative permittivity. The same charge density of 1 mC/m^2^ is assumed on the top and bottom surfaces while the generated electric field and the corresponding surface potential are calculated for a piezoelectric material with a thickness of 1 cm. Figure 2B shows a piezoelectric material at resonance surrounded by air and its corresponding voltage distribution, where the fringing electric field is represented by the black arrows. For materials such as Quartz, AlN, LiTaO_3_, and LiNbO_3_ with low/moderate relative permittivity, the electric field is higher than or very close to the air breakdown field (~ 3 MV/m), thus imposing a fundamental limit on the maximum radiation. Although one conceivable solution to this problem is non-metallic vacuum packaging, it increases both the antenna volume and cost, making it bulky and less reliable. On the other hand, an ADMIRE with a high relative permittivity such as PZT or PMN-PT (${\varepsilon }_{r}$> 1000) can be used to mitigate this issue. In addition to enabling greater maximum radiation, better near-field confinement inside high permittivity piezoelectrics results in the near-field region becomes shortened to a fraction of the distance compared to the near-field of an equivalent infinitesimal electric dipole^21^. To facilitate future material selection for optimal antenna performance, the following figure of merit for ADMIREs is defined:

9 $$FoM={d}^{2}{C}^{E}{\varepsilon }_{r}Q.$$

Orders of magnitude further improvement in radiation efficiency for acoustically driven antennas is expected with further optimization of material choice and design.

In addition to efficient radiation, passband transmission requires a modulation technique to send information. Simple and common digital modulation schemes can be utilized such as binary amplitude, frequency, and phase-shift keying (BASK, BFSK, BPSK) to directly modulate the ADMIRE (carrier) amplitude, frequency or phase with a modulating bitstream^25^. A mechanical antenna such as the ADMIRE has a settling time that is directly proportional to its quality factor and limits the BASK (on–off keying) rate since the mechanical system must be switched on and off corresponding to bit 1 and bit 0, respectively. The same applies to BPSK due to the phase discontinuity that requires the system to resettle and synchronize with the driving signal every time the phase changes. This presents a tradeoff between the material quality factor, which is required for efficient antenna radiation, and the maximum achievable data rate, which is required for bandwidth efficiency^25^. On the other hand, BFSK can be designed to have a fixed amplitude and continuous phase, sometimes referred to as continuous phase FSK (CPFSK), or minimum-shift keying (MSK), which mitigates the amplitude settling limitation but still has the same tradeoff as the bit rate in the case of BFSK is limited by frequency settling (different from amplitude settling) which is discussed in the results section.

An FoM presenting the characteristics of a BFSK modulator can be expressed as follows:

10 $${FoM}_{Mod}=\Delta f\times {FSK}_{Rate},$$

where $\Delta f$ is the separation between the two frequencies representing the binary message ($\Delta f={f}_{2}-{f}_{1}$) and ${FSK}_{Rate}$ is the maximum achievable FSK rate for switching between the two frequencies. For practical systems, $\Delta f$ must be as large as possible to allow for larger separation between the band-pass filters (BPF) in the receiver, which relaxes the BPF design specifications and reduces the bit error rate (BER), while higher ${FSK}_{Rate}$ enables higher bit rates (for BFSK ${Bit}_{Rate}=2\times {FSK}_{Rate}$).

### Design

**Figure 3:**
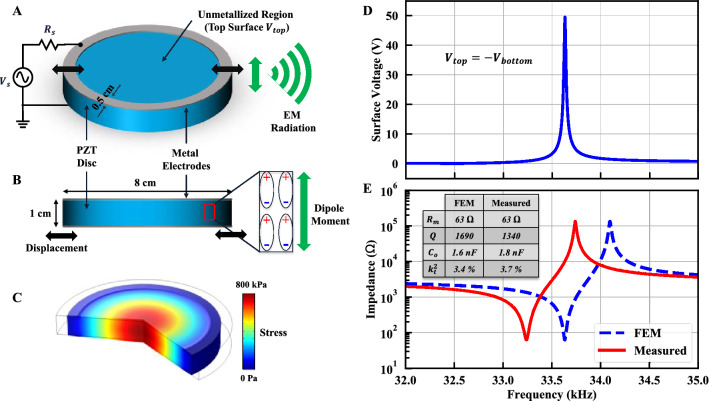
Proposed design and simulation of the piezoelectric radiating element using PZT. (**A**,**B**) The 3D view and the side view of the PZT disc (8 cm diameter, 1 cm thickness), with patterned silver electrodes along the disc circumference (0.5 cm width), driving source connected to the top and bottom electrodes, and dipole moment direction elaborated. (**C**) Resonance mode showing the radial stress distribution at resonance formed by the acoustic standing wave with an applied voltage amplitude of 1 V (simulated with COMSOL Multiphysiscs v4.4). (**D**) Simulated average surface voltage over the entire unmetallized region with an applied voltage amplitude of 1 V. (**E**) Simulated and measured impedances at the input terminals of the PZT disc.

Depending on the design goals, different resonance modes and frequencies can be targeted based on the piezoelectric material properties, dimensions, vibration direction, and excitation to meet performance metrics. In this paper, a high FoM ADMIRE antenna is designed to operate at the upper bound of the VLF band. Emphasis is placed on measuring the ADMIRE far-field radiation in the VLF band, and thus the FoM is constrained by frequency and geometry considerations and well below the ultimate FoM achievable for the ADMIRE. A disc resonator is designed with a high quality factor as shown in Fig. 3A,B, with 0.5 cm wide silver electrodes patterned around in a ring around the circumference of the PZT disc (top and bottom). This structure forms an acoustic resonator that is mechanically free with metal electrodes to drive it into resonance via the ${d}_{31}$ coefficient. The lateral vibration of the disc, also known as contour mode or dilation mode^26–28^, is excited by applying a time-varying voltage on the metalized edges of the PZT disc. Upon excitation, the time-varying electric field introduced by the electrodes (configured as a pair of top and bottom electrodes) excites the piezoelectric disc into vibration via the inverse piezoelectric effect. The excited acoustic wave is reflected by the PZT disc boundaries, resulting in a standing acoustic wave with its maximum stress at the disc center. Figure 3C shows the resonance dilation mode at 33.6 kHz along with the stress distribution.

During vibration, the mechanical stress generates electrical charges via the direct piezoelectric effect. The charges generated in the metalized electrode areas are neutralized by the electrodes, so the electrodes are designed around the edge of the disc where stress is lowest, leaving the highest stress, highest charge density center of the disc free to radiate. The density of the electrical charge is amplified by the quality factor at resonance, leading to a large time-varying dipole moment (current) that causes EM radiation. Additional geometries can be used to excite different high coupling piezoelectric materials in optimal resonant modes (such as dilation, thickness extensional or shear) to maximize generated charge, and thus radiation, due to higher piezoelectric coupling coefficients.

The structure is simulated using FEM. Figure 3D shows the generated surface voltage due to charge accumulation at resonance with an applied voltage amplitude of 1 V. A motional resistance of 63 Ω is designed to match with typical 50 Ω systems at the 33.6 kHz resonance, as seen in Fig. 3E which shows the impedance at the input terminals of the PZT disc (both simulated and measured).

Since ${R}_{rad}$ is negligible for matching consideration (${R}_{rad}\ll {R}_{m}$), the motional resistance can be further tailored for perfect matching with 50 Ω systems by changing the width of the electrodes to modify ${C}_{o}$. According to the BVD model ${R}_{m}$ can be expressed as shown in Eq. (4).

## Results

**Figure 4:**
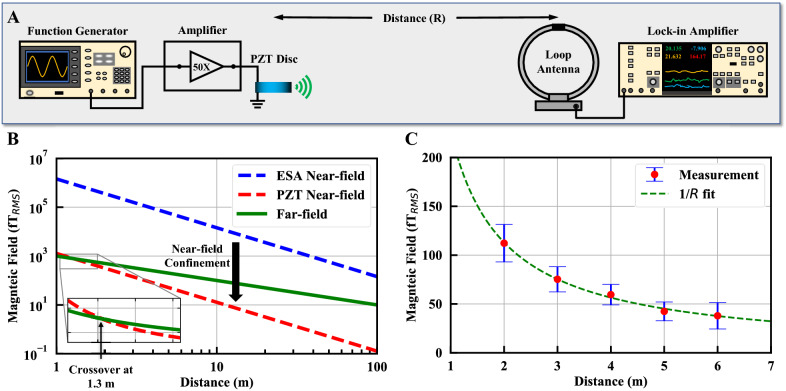
Simulation and measurement of the PZT radiation. (**A**) Measurement setup for detecting the magnetic field radiation of the ADMIRE. (**B**) Simulation comparing ESA (infinitesimal dipole) and PZT magnetic fields. Due to the high relative permittivity of PZT, the magnetic field is confined within it, which dramatically reduces the near-field component relative to the equivalent ESA approximation. The radiated far-field can be measured very close to PZT after passing the crossover point at 1.3 m. (**C**) Measured magnetic field vs. distance exhibiting clearly the far-field regime.

To demonstrate the ADMIRE antennas, a prototype is created from a 1 cm thick, 8 cm diameter disc of PZT. A 20 µm thick, 0.5 cm wide silver ring electrode is patterned onto the top and bottom surfaces and driven to excite the PZT in the dilation mode via the ${d}_{31}$ piezoelectric coefficient*.* The resonant response is extracted from a direct impedance measurement and yields the results shown in Fig. 3E. The radiation measurements of the ADMIRE are complicated by the near-field confinement due to the high permittivity of the PZT. Unlike the far-field radiation of the ADMIRE which is dependent only on the equivalent current caused by the flipping dipole moments, the radiated near-fields are confined by the large relative permittivity of PZT within the dielectric. This means that near-field radiation, characterized by 1/*R*^3^ for electric fields and 1/*R*^2^ for magnetic fields, is diminished in both magnitude and distance. Compared to an equivalently sized 33 kHz ESA which radiates in the near-field regime up to 1 km, the ADMIRE antenna reaches its far-field regime (magnetic) after around 1.3 m. Due to the respective distance scaling of 1/*R*^2^ vs. 1/*R*, equivalent magnetic field radiation from the ESA is 100 times larger at ten meters than the ADMIRE radiating the same power. Therefore, both the PZT disc and the measurement setup shown in Fig. 4A are designed to minimize RF interference from the leads and connections so that the PZT radiation is not obscured.

The magnetic field vs. distance is measured in free space to minimize RFI and scattering using the setup shown in Fig. 4A. As seen in Fig. 4C, the measured magnetic field decreases as 1/*R* as expected from the simulations in Fig. 4B, confirming the PZT ADMIRE exhibits far-field radiation very close to the antenna. An input power of 1.2 W is supplied to excite the PZT disc. Radiation is measured using a passive loop antenna and the magnetic field is extracted from a lock-in amplifier measurement using the measured antenna factor $AF={B}_{RMS}/{\mu }_{o}{V}_{RMS}$, where ${B}_{RMS}$ is the root mean square (RMS) magnetic field, ${V}_{RMS}$ is the voltage measured with the lock-in amplifier, and ${\mu }_{o}$ is the free space permeability. In order to better distinguish the measured radiation from noise, an average field reading is collected over two minutes at each distance. Extrapolating the measured data to 1 km yields a magnetic field of 0.23 fT_RMS_ with an ADMIRE driving power of 1.2 W compared to a simulated magnetic field of 0.5 fT_RMS._ The discrepancy between the simulated and measured field strengths is likely due to imperfect earth ground effects^14^, shifts in resonance due to ambient temperature changes, and effects from nearby radiators and reflectors.

**Figure 5:**
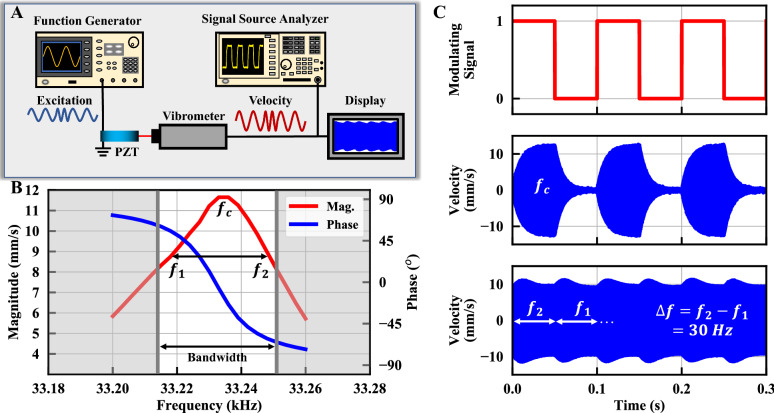
Different modulation schemes applied to the ADMIRE. (**A**) Mock-up of the measurement setup. (**B**) Frequency response of the velocity (both magnitude and phase are measured) at the PZT disc edge. (**C**) (top) 10 Hz bit stream, (middle) vibrometer velocity measurement of the amplitude-shift keyed (ASK) signal, and (bottom) vibrometer velocity measurement of the frequency-shift keyed (FSK) signal.

**Figure 6:**
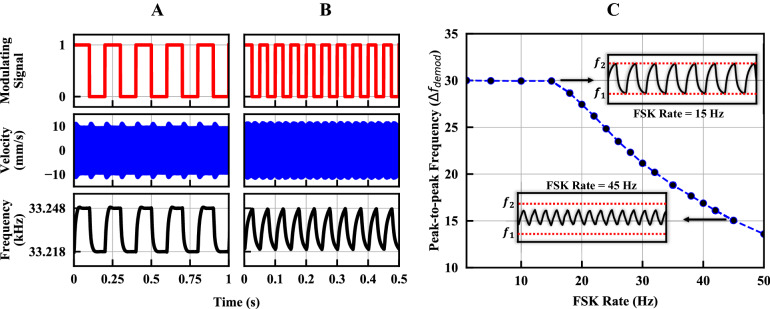
Measured FSK modulation of the PZT ADMIRE at different FSK data rates. (**A**,**B**) 5 Hz**,** and 20 Hz FSK rate. (**A**,**B**) (Top) modulating signal, (middle) measured velocity, and (bottom) demodulated signal using signal source analyzer (SSA). (**C**) Peak-to-peak frequency difference ($\Delta f_{demod} = f_{2} - f_{1}$) after demodulation with SSA at different FSK rates. The upper limit for direct modulation using the 3-dB bandwidth is limited by frequency settling, the maximum direct modulation frequency is approached resulting in a distorted modulation waveform.

The PZT disc is directly modulated using a function generator outputting both ASK and FSK signals with the resonant response of the PZT disc captured using an optical vibrometer as shown in Fig. 5A. In both cases the 10 Hz binary bit stream at the top of Fig. 5C is used. With the ASK signal, as the driving signal is switched on and off the resonator energy ramps up and down over a duration inversely proportional to the loaded quality factor (${Q}_{L}$ ≈ 850*)* The ramping time limits the fundamental modulation rate for direct BASK to approximately $1/2T$, where the time constant $T=3\times (2{Q}_{L}/\omega )\approx 24.4 ms$ (corresponding to 95% settling from the peak value) for the demonstrated measurement. A fundamental design tradeoff must be considered to balance the inversely proportional data rate with the high $Q$ desired for the radiation FoM. BFSK modulation is conducted within the 3-dB bandwidth of the PZT resonator corresponding to BFSK frequencies of ${f}_{1}$ = 33.218 kHz and $f_{2}$ = 33.248 kHz. The input is a continuous-phase FSK with no discontinuity when switching between the two frequencies. However, due to the phase difference of the mechanical resonator at the two frequencies, the mechanical resonance is out of phase with the modulated driving signal when it is switched and ramping of the PZT edge velocity occurs while energy is transferred from one resonant frequency to another as seen in Fig. 5C (bottom). As the modulation frequency approaches the limit set by the frequency settling, although the amplitude of resonance is not diminished, the demodulated output signal is distorted as seen in Fig. 6A,B. Multiple approaches can be implemented to surpass the *Q*-limited fundamental modulation rate of the resonator by ensuring that the phase of the resonator and driving signal are in phase during modulation transitions.

## Discussion

Despite the demand for portable VLF communication for long-range, low power applications with modest bandwidth requirements, electrical antennas have proven untenable at VLF frequencies due to the inherently poor tradeoff between electrical size and efficiency. Acoustic antennas operating at wavelengths up to five orders of magnitude less than EM wavelengths have been considered as alternatives to overcome the inefficiency of sub resonance operating sizes. However, until recently, piezoelectric materials have been insufficient to meet demands for handheld EM communication. As shown in Eq. (5), efficient radiation requires both large electromechanical coupling coefficients and a high mechanical quality factor, which is difficult to achieve since most piezoelectric materials empirically exhibit an inverse relationship between these parameters. Moreover, the relative permittivity of the piezoelectric material must be as high as possible in order to relax the electrical breakdown limit of the surrounding medium and maximize the radiated power. Additionally, even if desirable piezoelectric material properties can be achieved, fabricating the piezoelectric geometry and orientation to achieve the desired fundamental acoustic resonance mode can prove difficult due to poling and material growth considerations.

In this paper, a proof of concept VLF mechanical antenna, made of commercially available materials, devoid of any special packaging schemes, and surpassing the matched efficiency of ESAs by three orders of magnitude, is presented to lay a foundation for further enhancement. A bulk PZT disc which provides the best tradeoff between electromechanical coupling coefficients, mechanical quality factor, relative permittivity, and availability is demonstrated. The PZT dimensions are tailored to yield a fundamental acoustic resonance near the edge of the VLF range at 33 kHz as shown in Fig. 3E. Exciting the fundamental mode to align all the dipole moments inside the material in the same direction is paramount for maximum radiation as higher-order modes will have dipole moments aligned to opposite directions and partially cancel the EM radiation.

Unlike most electrical antennas where the far-field regime of VLF antennas occurs kilometers away from the source, far-field radiation from the ADMIRE can be measured as close as two meters. Typically, at distances less than a wavelength away from an antenna, the near-field reactive energy component is much larger than the far-field radiated energy. Since the reactive fields decay ($1/R$^2^) much faster with distance than the radiated fields ($1/R$), at some distance approximately one wavelength from the antenna the radiated field becomes larger than the reactive near fields. However, the high relative permittivity of the PZT (~ 1000) confines most of the reactive energy inside of the PZT, greatly reducing the cross-over point where the radiated fields become dominant and allowing the radiated far-field to be directly measured at distances as close as two meters. Although such a close far-field region is unconventional for VLF electrical antennas, the phenomena theoretically predicted by the FEM simulations shown Fig. 4B was experimentally verified as shown in Fig. 4C where the measured magnetic field decays at a rate of $1/R$. Since the reactive fields are normally much larger than the radiated fields near the antenna, the reduction of the reactive fields due to confinement inside of the PZT limits the currently achievable communication distance of the ADMIRE prototype for near-field communications. However, the high permittivity does not diminish the radiated far-fields. The effective current of the ADMIRE is extracted from COMSOL simulations in conjunction with the displacement profile measured with a Polytec OFV-5000 laser vibrometer. The expected magnetic field is then calculated from the effective current under the assumption that ADMIRE disk radiates like an infinitesimal dipole and is within 5% of the measured far-field magnetic field given in (3). Therefore, the high permittivity does not appear to diminish the radiated fields, and as more efficient materials, designs, and power handling schemes are implemented, scaling the link distance is much more promising than for ESAs.

To achieve orders of magnitude distance scaling without greatly increasing the size of transmitters, better designs are needed to harness the ultimate efficiency of piezoelectric antennas, which theoretically can be orders of magnitude greater than demonstrated here. In particular, higher coupling coefficient modes and materials provide significant promise for increasing efficiency. The demonstrated contour mode PZT ADMIRE is presented as a proof-of-concept and to lay the groundwork for piezoelectric antennas. From Eq. (5), maximizing resonator designs and materials with higher $d^{2} C^{E} Q$ products will lead to improvements in efficiency. For the PZT utilized for the ADMIRE, reconfiguring the volume to more efficiently excite the thickness resonant mode (via the ${d}_{33}$ coupling coefficient) could provide nearly an order of magnitude efficiency enhancement since ${d}_{33}\sim 3{d}_{31}$. Other materials such as PMN-PT with piezoelectric coupling coefficients more than 10 times higher than PZT have the potential for even more drastic efficiency enhancement^29^. Although the quality factors of commercially available relaxer ferroelectric materials such as PMN-PT are currently low (typically < 100), recent research on ion doping has shown promise to enhance Q in high-coupling ferroelectric materials such as manganese doped PMN-PZT^30^ (d = 1140 pC/N, C = 120 GPa, Q = 1050) which has been demonstrated with a ${d}^{2}{C}^{E}Q$ radiation efficiency product 45 times larger than the PZT demonstrated here. Although the moderate *Q*s limit the power handling due to increased heat dissipation, the total radiated power scales with radiation efficiency. Furthermore, the modest quality factors provide the potential for larger bandwidths, potentially enabling higher data rates and simpler frequency synchronization in piezoelectric antenna arrays. Modulation of the ADMIRE is demonstrated here with continuous phase BFSK in order to avoid amplitude settling. The directly modulated ADMIRE achieved modulation rates of up to 60 bps, which could potentially be increased to beyond 1 kbps in PMN-PT antennas without loss of efficiency. If much faster modulation rates are required, implementing additional circuitry such as reactive tuning elements to further decrease settling times or improving the receiver demodulation scheme can be adopted.

In summary, the presented ADMIRE demonstrates the potential for portable VLF transmitters that have been unattainable for decades. The framework provided here fully outlines the design space for ADMIREs and provides a roadmap to achieve orders of magnitude further improvement in radiation efficiency and modulation rate through the implementation of different vibrational modes and materials with high FoM. Although the current measurement distance is limited, the short standoff is primarily limited by power handling considerations and is not indicative of the ultimate communication distance of acoustic antennas. Thermal dissipation in the demonstrated ADMIRE limits the input power to be well below the mechanical (ultimate stress) or electrical (breakdown) driving limits. Improving temperature control to achieve greater power handling or implementing parallel arrays of ADMIREs, where phase synchronization is simplified by the moderate $Q$, can enable orders of magnitude improvement in the ADMIRE range. While this work is demonstrated at the upper end of the VLF range, using the provided theoretical framework and proposed FoM, this work can be applied to different frequencies by scaling the dimensions of the acoustic antennas. Since the operating frequency is determined by the length of the antenna along the resonant axis, increasing the operating frequency results in more compact antennas suitable for portable applications. The efficiency advantage over electrical state-of-the-art at both higher and lower frequencies can be maintained provided appropriate materials and designs are utilized, exhibiting great potential for numerous wireless IoT applications.

## Materials and methods

### Modeling

Piezoelectricity and EM radiation modeling require multidisciplinary understanding and coupling between the electrical and the mechanical domains. This is achieved by using FEM available from “COMSOL Multiphysics” that couples these domains in the “piezoelectric devices” toolbox. This toolbox solves the piezoelectric constitutive equations either in its stress-charge or strain-charge forms. Such a model can be used to determine the resonance frequency of the structure using eigenfrequency simulations followed by frequency domain simulations to find out parameters such as induced stress/strain, the velocity at the edge of the disc, internal polarization, surface voltages, and admittance. Two types of boundary conditions (BC) need to be set to run frequency-domain simulations; electrical BC and mechanical BC. Electrically, an arbitrary voltage amplitude is applied to the top electrode while the bottom electrode is grounded (all the parameters scales linearly with voltage) and all other surfaces are electrically floating. On the mechanical side, a free BC is assigned to the whole PZT disk to reduce any anchor damping and a material damping is assigned to PZT (see Supplemental Information for a detailed figure). The internal polarization (charge density) can then be used to calculate the polarization current and the radiated EM field from Eqs. (2) and (3). PZT piezoelectric properties are supplied by the vendor (see Supplemental Information) and input to the FEM model. The material damping (quality factor) is modified so that the motional resistance ${R}_{m}$, calculated using Eq. (4), matches the measured value. The simulation time can be dramatically reduced since our designed PZT disc exhibits symmetry around its central axis so, axisymmetric simulations are utilized. In addition, an air sphere is added to model the surrounding of the ADMIRE which enables near-field electrostatic simulations around the ADMIRE to compare air breakdown around different piezoelectric materials. The size of the air sphere is much smaller than the crossover point so that the radiated power is much smaller than the near-field reactive power and other losses in the device and can be neglected. Simulated admittance is compared to measured admittance in Fig. 3E. Moreover, the simulated near-field of PZT is compared with the infinitesimal dipole near-field in Fig. 4B.

### PZT fabrication and characterization

The PZT discs are commercially fabricated by Physik Instrumente (PI) and made from their PIC181 material^31^. It is a hard PZT chosen to balance the mechanical quality factor and the electromechanical coupling. The commercial discs have an 8 cm diameter and a 1 cm thickness with both top and bottom surfaces fully metalized with ~ 20 µm of silver. Patterning of the silver is conducted using an end mill to remove the interior metal until only the desired 0.5 cm ring along the edge remains. Two wire leads are split from a BNC cable and soldered to the top and bottom metal surfaces to provide electrical excitation, with the lead lengths minimized to reduce near-field radiation from the current loop.

During testing, the PZT disc operates with mechanically free boundary conditions, such that all moving surfaces are free to move without constraint which theoretically results in the lowest damping and highest $Q$. The mechanically free boundary condition is achieved by resting the bottom center of the resonator on a small piece of foam. In theory, the center of the disc where the lateral displacement is zero (nodal point) is the optimal contact point. The lack of motion results in negligible surface friction and thus does not affect the resonance mode. Multiple mounting configurations were considered, with the small foam base ultimately chosen for simplicity as it was experimentally verified to not reduce resonator $Q$ and resonator quality factor approached the theoretical limit set by the manufacturer.

Characterization of the PZT is conducted by connecting a Tektronix AFG3152C function generator directly to the electrodes via BNC cable. An Agilent E4445A spectrum analyzer connected in series with the PZT disc is then used to characterize the impedance of the PZT. The spectrum analyzer measures the power spectrum as a function of frequency which is then used to calculate the magnitude of the ADMIRE impedance. The measured power spectrum is then fit using the BVD model from which the motional resistance, electromechanical coupling (${k}_{t}^{2}$) and mechanical quality factor are extracted. The bottom surface of the PZT disc rests freely on a 2 × 2 cm insulating cardboard lattice and the top and side surface are unconstrained. Multiple clamping configurations were considered but yielded negligible changes in mechanical properties. Input power to the PZT disc is characterized by removing the series spectrum analyzer and adding an Agilent MSO7104B oscilloscope in parallel with the disc with the power dissipation measured from the voltage drop across the PZT.

### Radiation measurement

Wireless radiation measurements of the generated magnetic field are conducted in an open environment to minimize scattering and noise. Confinement of the near-field component of the PZT radiation results in the far-field component dominating beyond 2 m but current loops in the transmitter exhibit near-field dominate radiation up to 1000 m. In order to minimize near-field radiation from current loops, leads and connections are minimized and oriented to exhibit radiation orthogonal to the receiving antenna. The resulting total radiation exhibits a near-to-far-field crossover between 1 and 2 m. At the operating frequency, only the PZT radiation can exhibit a $1/R$ roll off at distances less than 1 km, therefore all measured radiation with a $1/R$ fit is attributed solely to the PZT.

The transmitting system consists of a Tektronix AFG3152C function generator connected in series to a 50 × Trek model 2100HF amplifier to generate a sufficiently large excitation to measure the far field. The amplifier presents a resistance of 200 Ω in series with the 63 Ω motional resistance of the PZT disc at resonance which results in a diminished loaded quality factor where ${Q}_{L}=Q{R}_{m}/({R}_{m}+{R}_{s})$. From Eq. (5), the diminished ${Q}_{L}$ results in a lower radiation efficiency for the PZT and a higher power is needed to drive the loaded ADMIRE.

The magnetic field is measured using an AH-Systems SAS-565L 24″ shielded passive loop antenna which is oriented to receive the maximum signal from the PZT far-field component. Incident radiation induces an open circuit voltage across the antenna terminals proportional to the field strength. The antenna factor of the loop receiver is calibrated by the manufacturer post-production to be 1.74 Ω^−1^ m^−1^ at 33 kHz and is used to extract the measured B-field where ${B}_{RMS}=AF{\mu }_{o}{V}_{RMS}$. The open-circuit voltage of the antenna is measured using a Stanford Research Systems SR865A lock-in amplifier that is frequency locked to the transmitting PZT disc and employs a 24 dB/octave bandpass filter to attenuate noise around the locked frequency. Measurements were made at 1 m distance intervals for 2 min at a time using a 1 s time constant. The measured B-field strength is extracted from the average terminal voltage over the 2-min measurement window, with 1 standard deviation error bars also provided to account for variance in the measured field strength due to noise. Between field measurements, the noise floor is measured at 1-min intervals with the input signal turned off (Supplementary Fig. 3). Measurements beyond 6 m exhibit a signal-to-noise ratio < 2 and are not shown.

### Modulation measurement

Direct digital modulation of the ADMIRE can be done by altering amplitude, frequency, and phase of the excitation signal which in turn modulates the mechanical resonance of the ADMIRE and thus radiated signal. In this paper, we focus on BFSK since it has a continuous phase which lowers the mechanical settling time compared to both BPSK and BASK. The modulation is evaluated using the measurement setup in Fig. 5A. The setup consists of a Tektronix AFG3152C function generator that directly excites the ADMIRE with continuous phase BASK or BFSK signals. A Polytec OFV-5000 laser vibrometer is used to measure the velocity of the PZT edge while vibrating in the dilation mode. The two BFSK modulation frequencies are chosen to be within the 3-dB bandwidth as shown in Fig. 5B. Figure 5C shows the modulated velocity of BASK and BFSK with a 10 Hz modulation rate. The velocity signal is then input to a ROHDE and SCHWARZ FSUP signal source analyzer with FM demodulation capability to demodulate the signal as shown in Fig. 6A,B (bottom figures) for 5 Hz and 20 Hz BFSK rates, respectively. Moreover, the peak-to-peak frequency difference after demodulation at different FSK rates is shown in Fig. 6C.

## Supplementary information

Supplementary Information.

## Data Availability

All data needed to evaluate the conclusions in the paper are present in the paper and/or the Supplementary Materials. Additional data available from authors upon request. Requests for materials should be addressed to A. E. Hassanien (email: ahmedeh2@illinois.edu). All figures in the manuscript and Supplementary Materials are prepared by the authors. Software packages used to prepare the figures are: Microsoft Powerpoint (URL: https://products.office.com/en-us/powerpoint), Python (URL: https://www.python.org/), and COMSOL Multiphysics v4.4 (URL: https://www.comsol.com/).
