# Supplementary material for: Acoustically driven electromagnetic radiating elements
Source: Sci Rep. 2020 Oct 12;10:17006. doi: 10.1038/s41598-020-73973-6 (PMC7550615; doi:10.1038/s41598-020-73973-6)
Supplement: Supplementary file 1 — Supplementary Information. [file 41598_2020_73973_MOESM1_ESM.pdf]

Acoustically Driven Electromagnetic Radiating Elements

Ahmed E. Hassanien[1], Michael Breen[1], Ming-Huang Li[2] & Songbin Gong[1]

[1] University of Illinois at Urbana-Champaign, Department of Electrical and Computer Engineering, Micro and Nanotechnology Laboratory, Urbana, IL, 61801.

[2] National Tsing Hua University, Department of Power Mechanical Engineering, Taiwan 30013.

Correspondence and requests for materials should be addressed to A. E. Hassanien (email: ahmedeh2@illinois.edu)

Supplementary Information:

1. Antenna Matched efficiency

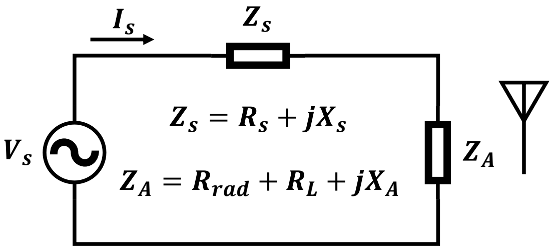

Fig. S1. General antenna model of a transmitter. Equivalent circuit model used to derive the matched total antenna efficiency of the ADMIRE relative to an equivalent ESA based on an infinitesimal dipole. Z_A is the antenna complex impedance and Z_s is the source complex impedance.

Matched total antenna efficiency is a measure of both how efficiently power can be delivered from a source to the antenna and how well the antenna radiates the power delivered to it:

$$\xi_{tot} = \xi_{\Gamma}\xi_{rad} = \frac{P_{del}}{P_{av}}\frac{P_{rad}}{P_{del}} = \frac{P_{rad}}{P_{av}} \quad (1)$$

Therefore the matched total efficiency of the antenna, ξ_{tot}, is defined as the product of the mismatch efficiency, ξ_{Γ}, and the radiation efficiency, ξ_{rad}, where P_{av}, P_{del}, P_{rad} are the power available from the source, power delivered to the antenna and power radiated by the antenna respectively.

The radiated power from an antenna is defined as:

$$P_{rad} = \frac{|I_s|^2 R_{rad}}{2} = \frac{|V_s|^2}{2}\left(\frac{R_{rad}}{(R_{rad} + R_L + R_s)^2 + (X_A + X_s)^2}\right) \quad (2)$$

where the circuit elements are shown in Fig. S1. Maximum radiated power occurs for the case of a lossless antenna ($R_L = 0$) with a conjugate match between the source and antenna impedances ($Z_A = Z_s^*$) such that all the power available from the source (50%) is delivered to the antenna and 50% of the power is dissipated in the source resistance. This maximum theoretical limit is the same for both ADMIRE and ESA antennas using the same source.

The ADMIRE antenna is matched so that $X_A = X_s = 0$ and the antenna losses are represented by the motional resistance ($R_L = R_m$). Equation (2) can be modified for the ADMIRE as follows:

$$P_{rad}^{ADMIRE} = \frac{|V_s|^2}{2}\left(\frac{R_{rad}^{ADMIRE}}{(R_{rad}^{ADMIRE} + R_m + R_s)^2}\right) \quad (3)$$

On the other hand, infinitesimal dipole antennas require matching to cancel out the large reactive part that occurs in non-resonant ESAs. In that case $X_A = -X_s$ and $R_L = R_{loss} + R_{match}$ where R_{loss} represents the ESA conduction/dielectric losses and R_{match} is the loss due to the finite quality factor of the matching element. Equation (2) can be modified for ESAs as follows:

$$P_{rad}^{ESA} = \frac{|V_s|^2}{2}\left(\frac{R_{rad}^{ESA}}{(R_{rad}^{ESA} + R_{loss} + R_{match} + R_s)^2}\right) \quad (4)$$

ESA is used here for comparison. Using all previously stated assumptions and dividing (3) by (4), the matched total antenna efficiency ratio, which is the ratio of radiated power from ADMIRE to radiated power from ESA using the same source, is expressed as:

$$\xi_{tot}^{rel} = \frac{\xi_{tot}^{ADMIRE}}{\xi_{tot}^{ESA}} = \frac{P_{rad}^{ADMIRE}}{P_{rad}^{ESA}} = \frac{R_{rad}^{ADMIRE}}{R_{rad}^{ESA}} \frac{(R_{rad}^{ESA} + R_{loss} + R_{match} + R_s)^2}{(R_{rad}^{ADMIRE} + R_m + R_s)^2} \quad (5)$$

The matched efficiency boost of the ADMIRE antenna is calculated using equation (5) in conjunction with equations (6)-(13).

$$R_{rad}^{ADMIRE} = \frac{B_{rad}}{\eta^2} \quad (6)$$

$$B_{rad} = 320\pi^2 \left(\frac{d_{ij} C_{jj}^E A_{PZT}}{\lambda}\right)^2 \quad (7)$$

$$\eta^2 = 4\frac{C_{jj}^E A_{PZT}}{L_{PZT}} C_o k_t^2 \quad (8)$$

$$R_m = \frac{\pi^2}{8\omega_r C_o k_t^2 Q} \quad (9)$$

$$R_{rad}^{ESA} = 80\pi^2 \left(\frac{L_{ESA}}{\lambda}\right)^2 \quad (10)$$

$$R_{match} = \frac{|X_A|}{Q_{inductor}} \quad (11)$$

$$X_A = -\frac{120\lambda}{\pi L_{ESA}}\left[ln\left(\frac{L_{ESA}}{2r_{ESA}}\right) - 1\right] \quad (12)$$

$$R_{loss} = \frac{L_{ESA}}{\sigma_c A_{ESA}} \quad (13)$$

Where B_{rad}, η^2 are the mechanical damping coefficient and the electromechanical coupling coefficient of the ADMIRE antenna respectively, $\omega_r = 2\pi \times 33$ kHz is the resonant frequency for both antennas and λ is the free space wavelength of the electromagnetic radiation. The piezoelectric coefficient, d_{ij}, and stiffness constant, C_{jj}^E, are given generally in (7) since different, or even multiple, piezoelectric coefficients and stiffness constants can be used to generate a magnetic field. For the ADMIRE PZT disc demonstrated here operating in the dilation mode, analytical calculations indicate, and FEM simulations confirm, that d_{31} and C_{11}^E are the dominant contributors to radiation. The ESA is assumed to be made from copper with bulk conductivity σ_c and matched with an inductor with a Q of 200. Using (6)-(13) in conjunction with Supplementary Table 1 and R_s=50 Ω, yields an improvement in matched antenna efficiency of more than 6400x.

The previous discussion assumes the same source for both ADMIRE and ESA with the same source resistance and same available power for radiation where the matched efficiency is defined as the ratio of power radiated to the maximum power available for radiation. An alternative way to define the matched efficiency ratio would be to consider defining the matched total efficiency as the radiated power to input power ratio assuming different power sources are available that can match each type of antenna separately. In that case equation (5) can be modified as follows:

$$\xi_{tot}^{rel} = \frac{R_{rad}^{ADMIRE}}{R_{rad}^{ESA}} \frac{(R_{rad}^{ESA} + R_{loss} + R_{match} + R_{s}^{ESA})}{(R_{rad}^{ADMIRE} + R_{m} + R_{s}^{ADMIRE})} \quad (6)$$

which results in 56x matched antenna efficiency improvement if compared with ESA, where $R_s^{ESA} = R_{rad}^{ESA} + R_{loss} + R_{match}$ and $R_s^{ADMIRE} = R_{rad}^{ADMIRE} + R_m$.

The ADMIRE antenna parameters are either characterized by the manufacturer (*) or measured post-fabrication.

ADMIRE Parameters

d_{31}	C_{11}^{E}	r_{PZT}	A_{PZT}	L_{PZT}	k_t^2	C_o
108* pC/N	152.3* GPa	3.5 cm	πr_{PZT}^2	1 cm	3.7%	1.8 nF

ESA Parameters

	L_{ESA}	r_{ESA}	A_{ESA}	σ_c	$Q_{inductor}$	
	8 cm	0.5 cm	πr_{ESA}^2	5.9×10^7 S/m	200	

Table S1. ADMIRE and ESA Parameters for Matched Efficiency Calculation

2. PZT material properties

The PZT discs (PIC-181) were bought commercially from PI ceramics (www.piceramic.com). Relevant material properties for the ADMIRE design are given below. The demonstrated PZT ADMIRE utilizes $\boldsymbol{d_{31}}$ for proof of concept and is far from the conceivable limit for acoustically driven antennas. As seen from (7), incorporating different $\boldsymbol{d}_{ij}$ provides the potential for a further order of magnitude improvement in radiation efficiency.

$$\varepsilon_r^T = \begin{bmatrix} 1224 & 0 & 0 \\ 0 & 1224 & 0 \\ 0 & 0 & 1135 \end{bmatrix}$$

$$\varepsilon_r^S = \begin{bmatrix} 740 & 0 & 0 \\ 0 & 740 & 0 \\ 0 & 0 & 624 \end{bmatrix}$$

$$d = \begin{bmatrix} 0 & 0 & 0 & 0 & 389 & 0 \\ 0 & 0 & 0 & 389 & 0 & 0 \\ -108 & -108 & 253 & 0 & 0 & 0 \end{bmatrix} pC/N$$

$$e = \begin{bmatrix} 0 & 0 & 0 & 0 & 475 & 0 \\ 0 & 0 & 0 & 475 & 0 & 0 \\ -120 & -120 & 265 & 0 & 0 & 0 \end{bmatrix} C/m^2$$

$$S^E = \begin{bmatrix} 11.75 & -4.07 & -4.996 & 0 & 0 & 0 \\ -4.07 & 11.75 & -4.996 & 0 & 0 & 0 \\ -4.996 & -4.996 & 14.11 & 0 & 0 & 0 \\ 0 & 0 & 0 & 35.3 & 0 & 0 \\ 0 & 0 & 0 & 0 & 35.3 & 0 \\ 0 & 0 & 0 & 0 & 0 & 31.6 \end{bmatrix} pm^2/N$$

$$C^E = \begin{bmatrix} 152.3 & 89.09 & 85.47 & 0 & 0 & 0 \\ 89.09 & 152.3 & 85.47 & 0 & 0 & 0 \\ 85.47 & 85.47 & 134.1 & 0 & 0 & 0 \\ 0 & 0 & 0 & 28.3 & 0 & 0 \\ 0 & 0 & 0 & 0 & 28.3 & 0 \\ 0 & 0 & 0 & 0 & 0 & 31.61 \end{bmatrix} GPa$$

3. Lock-in Radiation Measurements

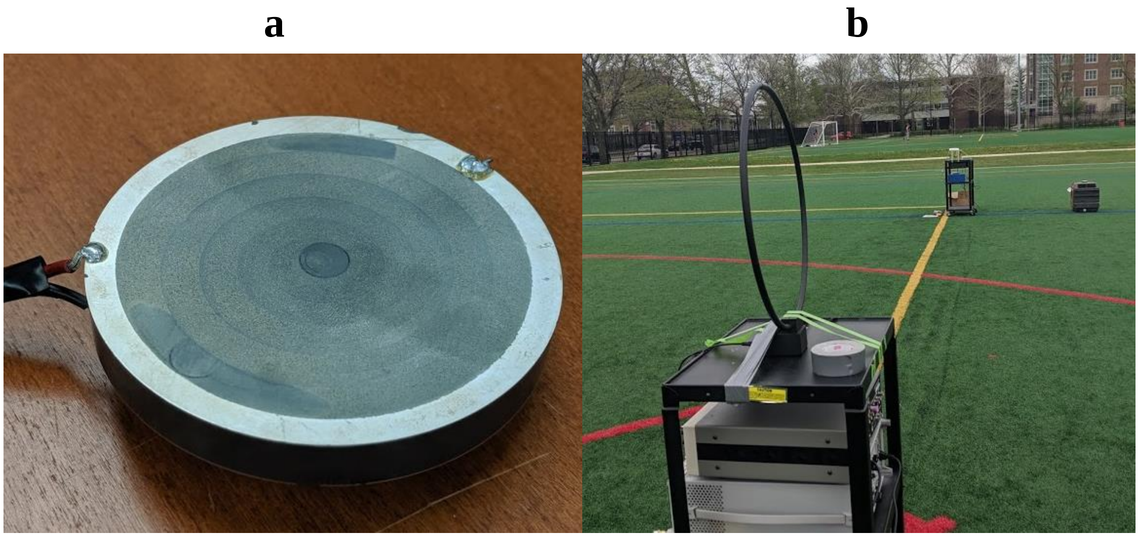

Fig. S2. Measurement of the ADMIRE. a, Fabricated PZT ADMIRE with leads kept short and close to minimize radiation from the current loop. **b**, Field measurement in an open athletic field to reduce scattering and background noise. The transmitter (consisting of the function generator, amplifier, and the ADMIRE) is powered separately from the receiver (consisting of the passive loop antenna and lock-in amplifier) using gas generators to remove ground loop radiation (Photo Credit: Michael Breen, University of Illinois at Urbana Champaign).

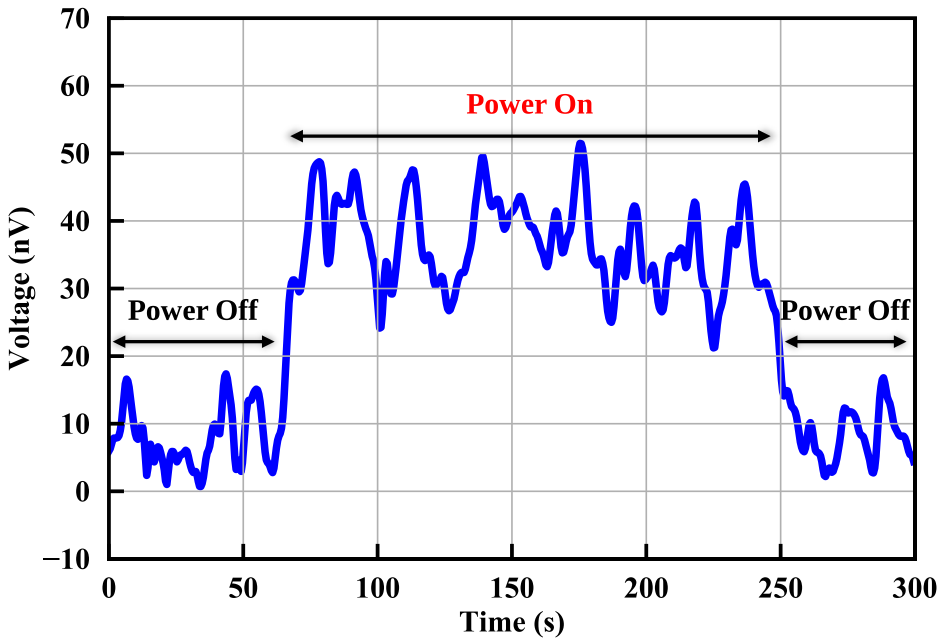

Fig. S3. Sample of measured voltage from lock-in amplifier vs. time at 2 meters. The measured voltage from the antenna terminals from which the measured magnetic field is extracted using the antenna factor. Both the field measurements (power on) and noise (power off) are post-processed and extracted. Average noise floor < 10 nV.

4. Spectrum Measurements and Mechanical Response

The below figure shows the magnetic field frequency response at 1m (indoors) that is radiated by the PZT disk measured with a loop antenna connected to a spectrum analyzer while at the same time the disk edge velocity is measured using an optical interferometer. The figure shows a complete correlation between the two frequency responses. The used drive voltage = 25 V peak-to-peak and the frequency sweep range is limited by the noise floor of the spectrum analyzer

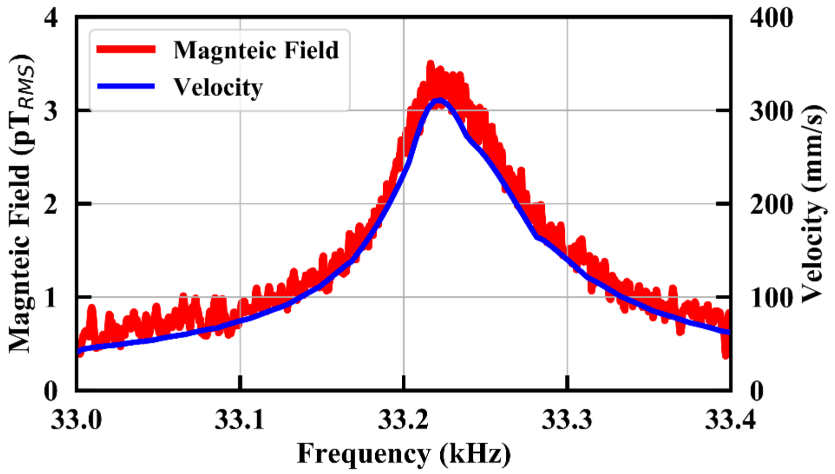

Fig. S4. Magnetic field and velocity frequency responses. The magnetic field frequency response at 1m (indoors) measured with a loop antenna connected to a spectrum analyzer. At the same time the disk edge velocity is measured using an optical interferometer (drive voltage = 25 V pk-pk).

5. Simulation Region:

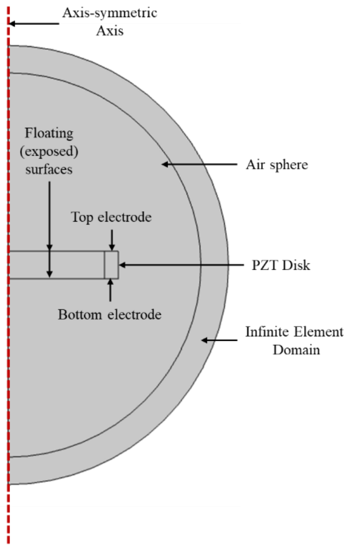

Fig. S5. Axis-symmetric simulation region. Air around PZT is represented as a half circle. An arbitrary voltage amplitude is applied to the top electrode while the bottom electrode is grounded

and all other surfaces are floating. A free mechanical boundary condition is assigned to the whole PZT disk to reduce any anchor damping.

6. Further Analysis on Additional Current Elements:

There are additional significant currents that need to be explained to show that the intended radiating element is the only element that is being measured. There will be large components of radial currents but these currents will cancel each other as they have the same magnitude and opposite direction. This is clarified in the following figure that show the surface voltage and radial current distribution on the cross-section of the Disk:

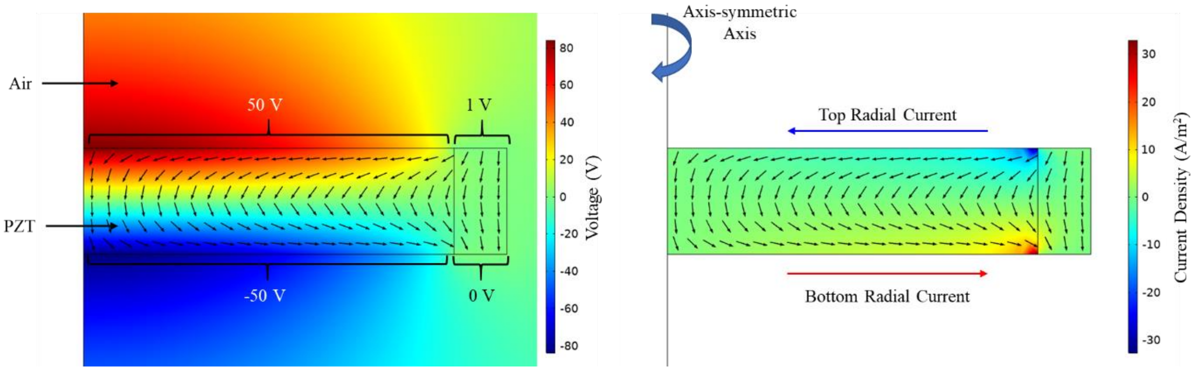

Fig. S6. Additional current elements simulation. the black arrows show the direction of displacement current within the PZT cross-section. The simulation is Axis-symmetric

As shown in the figure the top and bottom radial currents cancel each other, the black arrows show the direction of displacement current within the PZT cross-section. Moreover, due to the axis-symmetry nature of the disk (imagine the cross-section is revolved 360° around the axis-symmetric axis), all the radial components cancel and the only remaining current will be the thickness directed component.

The current flowing in the leads is opposite in direction and ~20% in magnitude of the current in the exposed region. For the simulated case, the terminal current from the source equals the applied voltage divided by the motional resistance ~ 16 mA (confirmed by simulation), the polarization current in the exposed region is ~ 82 mA (confirmed with supplied equations in the manuscript and simulated using the voltage or electric field within the exposed region) as shown in the previous figure where the surface voltage amplified by the quality factor reaches an average of ±50 V on the top/bottom exposed surfaces. This results in a 20% reduction in measured magnetic. The previous discussion assumes that the current in the leads contributes as a dipole not as a loop because in our measurement setup we reduce the loop area and put it in an orientation that generates a magnetic field that is orthogonal to the receiver loop used to measure the magnetic field. This can be confirmed by the magnetic field measurement done that has 1/R magnetic field decay, which can never be due to the loop as theoretically loops have 1/R3 magnetic field decay in the near-field region.

With all previous considerations in mind, we assume that the intended radiating element is the only element that is being measured.

7. Magnetic Field Calculation from Electrostatic Simulations:

Electric and magnetic field components for infinitesimal electric dipole radiation can be written as follows:

$$E_\theta = \eta \frac{jkq\omega d}{4\pi r} \sin\theta \left(1 + \frac{1}{jkr} - \frac{1}{(kr)^2}\right) e^{-jkr} \tag{14}$$

$$H_\varphi = \frac{jkq\omega d}{4\pi r} \sin\theta \left(1 + \frac{1}{jkr}\right) e^{-jkr} \tag{15}$$

The wave impedance in air can be calculated and plotted as follows:

$$\eta = \frac{|E_\theta|}{|H_\varphi|} \tag{16}$$

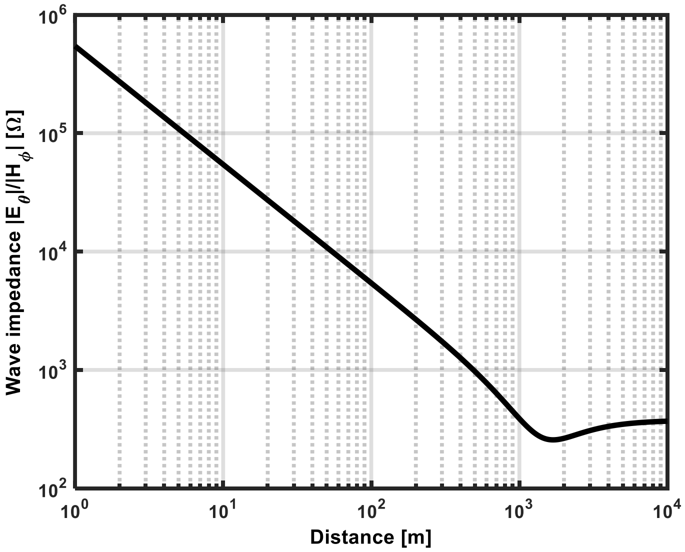

Using the simulated near-field electric field and calculated wave impedance in air we can calculate the corresponding magnetic field. Note that, the main assumptions here are:

1. Wave impedance is a material property and doesn't change if the material is fixed. The surrounding material is always air and the relative permittivity of the transmitter is the one that changes. So, by simulating the near-field in the air region and knowing the wave impedance the corresponding magnetic field can be calculated.
2. The far-field component is only dependent on the current so changing the permittivity of the transmitter does not change the far-field as long as the current is fixed. The far-field is the first term in the brackets of (14) and (15).
